# Electrical analysis of logical complexity: an exploratory eeg study of logically valid/invalid deducive inference

**DOI:** 10.1186/s40708-023-00194-8

**Published:** 2023-06-07

**Authors:** Francisco Salto, Carmen Requena, Paula Alvarez-Merino, Víctor Rodríguez, Jesús Poza, Roberto Hornero

**Affiliations:** 1 Grupo Neurociencia, Envejecimiento y Lógica Aplicada, Departamento de Psicología, Universidad de León, Campus Vegazana s/n, 24071 León, Spain; 2 Centro de Investigación Biomédica en Red, Universidad de Valladolid, Campus M. Delibes, Paseo Belén 15, 47011 Valladolid, Spain

**Keywords:** Beta-2 band, Evoked potentials, Induced potentials, Deductive inference, Logical validity, Cortical bases of logical reasoning

## Abstract

**Introduction:**

Logically valid deductive arguments are clear examples of abstract recursive computational procedures on propositions or on probabilities. However, it is not known if the cortical time-consuming inferential processes in which logical arguments are eventually realized in the brain are in fact physically different from other kinds of inferential processes.

**Methods:**

In order to determine whether an electrical EEG discernible pattern of logical deduction exists or not, a new experimental paradigm is proposed contrasting logically valid and invalid inferences with exactly the same content (same premises and same relational variables) and distinct logical complexity (propositional truth-functional operators). Electroencephalographic signals from 19 subjects (24.2 ± 3.3 years) were acquired in a two-condition paradigm (100 trials for each condition). After the initial general analysis, a trial-by-trial approach in beta-2 band allowed to uncover not only evoked but also phase asynchronous activity between trials.

**Results:**

showed that (i) deductive inferences with the same content evoked the same response pattern in logically valid and invalid conditions, (ii) mean response time in logically valid inferences is 61.54% higher, (iii) logically valid inferences are subjected to an early (400 ms) and a late reprocessing (600 ms) verified by two distinct beta-2 activations (*p*-value < 0,01, Wilcoxon signed rank test).

**Conclusion:**

We found evidence of a subtle but measurable electrical trait of logical validity. Results put forward the hypothesis that some logically valid deductions are recursive or computational cortical events.

**Supplementary Information:**

The online version contains supplementary material available at 10.1186/s40708-023-00194-8.

## Introduction

Even though the study of deduction is one of the oldest sciences, it is still a current source of challenges and open problems for contemporary cognitive neuroscience. While the formalistic validity-centered approach to deductive phenomena has been overcome [1, 2], the underlying neural processes associated to valid deductions is still an open issue. This paper focus on propositional logically valid truth functional arguments which are also probabilistically valid in Adam’s sense [3]. At an abstract computational or normative level [4, 5] these arguments demonstrably preserve the truth, probability and demonstrability of their inputs whichever content they may have [6]. In this regard, one crucial question is: are there any neural patterns characterizing logically valid deductions as inferential events? When arguments are not considered as abstract relations among propositions or probabilities, but as cortical time-consuming events, are deductive inferences genuinely distinct? Two opposing trends make this question relevant for neurocognitive science. On the one hand, if there are valid deductions, some neural events literally (not only metaphorically) compute inferential conclusions. On the other hand, there may be no valid deductions at all as cortical events, as suggested by the inconstant [7, 8] and weak [9] psychometrical evidence for the distinction between deductive and inductive inferences. From this perspective, the existence of valid deductions as cortical phenomena is not presupposed, opening up the methodological chance to verify or refute them.

In the last 20 years, research on the neural basis of deductive reasoning has achieved relevant conclusions. The first generation of experimental results was systematized by Knauff [10, 11] Goel [12] and Prado [13, 14] showing how different neural networks corresponded to propositional, relational, or categorical deductive inferences. The ulterior generation of studies focused on relational [15] propositional [16] or categorical [17] reasoning, where spatial and linguistic neural circuits are distinguished according to the type of inference and not according to the format of the stimuli (visual, linguistic, or agentual). A proper understanding of neural dependencies between different types of inference and formats is still lacking, even if the last reviews [18, 19] and metanalyses [17, 20] contribute to systematize the complex functional relations between the neural correlates of linguistic and deductive processes. Moreover, research has determined the presence in valid deductive reasoning [16] of double processing or re-processing in a late temporal phase. This temporal retardment phenomenon has been also verified in inhibitory control tasks [21] and after logical training [22, 23].

Neural analyses of propositional inferences have focused on premise-conclusion integration, which consists of premises and conclusions literally sharing variables [12, 24, 25]. Both linguistic terms and visual elements are shared in integrable inferential processes, for example, from the premises {(if ∙, then ♣), ∙}, it is integrable to deduce ♣, but not integrable to deduce ♥ or other unrelated or irrelevant conclusions such as “Hong Kong is in Asia”. Research on the spatial cerebral [12, 24, 26, 27] and temporal neuroelectrical [28–32], dynamics of these processes has proven the neural impact of integration and has located its processing partially in typically linguistic areas of the brain [33]. From a neuroelectrical perspective, previous research on propositional inferences has revealed significant attentional (P200, N250), premise integration (P300), semantic processing (N400) and late reprocessing (P600) ERPs. In linguistically codified inferences, such as conditional or categorical inference, P200 is associated with selective attention and attentional demand [34, 35] with increased presence in non-integrable premisses. However, in visual inferences, the attentional component is rather N250 [36, 37]. P3 ERPs have been reported to be associated with information monitoring, cognitive control and memory updating [38, 39] and in particular the presence of this component in human reasoning premise integration has been proven in [30, 31] The P600 component is associated with syntactic analysis and syntactic rule following [40, 41]. In the case of logical reasoning, research has observed enhanced P600 amplitudes attributed to reprocessing [16, 42]. In contrast with P300 and P600, the N400 component is generally associated with semantical content processing both in linguistic and visual settings [43, 44]. A complete picture of relevant neuroelectrical techniques in this study includes time–frequency analysis, in which power and phase information in the EEG signal are separated across different frequencies thus obtaining crucial data about the oscillations contained in the EEG signal [45, 46]. The literature shows that both logically valid and invalid propositional reasoning involve left frontoparietal circuits. Also, in simple valid deductive inferences (such as Modus Ponens and Disjunctive Syllogism), the neural processing is determined by relational complexity and not by logical complexity. From this integrative approach to deduction, we may interpret that valid and invalid deduction are developed over the same neural substrate, which basically depends on the semantic content and not on the logical structure of the information. This is not an isolated cognitive discovery, but a consistent trend correcting previous formalistic approaches to deduction [47]. Nevertheless, research has not yet developed tools for the measurement of integration in visual inferences (see [48] for a proposal). More importantly, premise integration excludes a wide family of deductive inferences which are valid, but not integrable. For example, logically valid inferences sharing no common content, such as arbitrary propositions validly deduced from contradictions, or such as tautologies validly deduced from adventitious propositions. These are in fact eventually taken as control or baseline in deductive reasoning experiments because their conclusions are not integrable with their premises. In this regard, integration does not offer a single neural support for deductive and non-deductive inferences since valid deductive inferences are excluded.

A second research line has focused on studying the complexity and not on integration of the neural correlates of deduction. Logical complexity is the number of occurrences of logical operators in a cognitive task, while the relational complexity is the number of variables or memory load of that cognitive task. The strategy of Monti [37, 50, 51] and [52, 53], is to study the neural effect of increasing logical complexity in reasoning tasks. In this regard, the cerebral correlates of logical complexity are experimentally identified and dissociated from semantic content processing. The research methodology has been progressively refined over the years and, as a result, a wide range of studies has shown the implication of specifically deductive “core” frontal areas (including both the mesial Brodmann Area 8, BA8, and the left rostrolateral prefrontal cortex, BA10), which do not match with the linguistic areas identified by the integrational perspective [52, 54]. Our experimental setting is designed to measure the neuroelectrical balance between content and logical complexity and thus eventually bringing together these two research lines.

In order to tackle the methodological challenge involved in making logical validity a measurable cortical magnitude, it is essential to recall the existence of metamathematical and probabilistic measures of validity which are complete, precise, and decidable [6]; although they are abstract computational procedures, which do not directly correspond to any spatio-temporal process in the brain. Cognitive science has searched for factive deductive measures that were experimentally viable, as the early works of Rips [55] and Wilhelm [56] show. Heit and Rotello [57] proposed the ratio “number of words/impact of validity” to distinguish deductive from non-deductive inferences. In the field of neuroscience, two indexes have been proposed and employed by Reverberi et al. [27, 58] associating physical magnitudes with logical validity. Other researchers [42] have systematized semi-recursive deductive measures corresponding to logical operators such as order, identity and repetition. The new experimental paradigm presented in this paper avoids any need to measure relational complexity, because it remains fixed or unaltered along the experiment. Logical complexity is measured in the usual way with the number of occurrences of logical operators [59].

The number and content of relational variables determines variations in the non-logical cognitive load of the tasks (relational complexity of the task), while the number of logical operators determine variations in the logical complexity of inferences (logical complexity of the task). The new experimental paradigm here presented fixes the same set of relational variables for valid (deductive) and invalid conditions, while only in the valid condition an increase in logical complexity appears. The objective of this study is to verify or refute the following hypothesis: there are specific EEG measurable neural activity patterns associated with logically valid deductive inferences. Methodologically, the new paradigm is designed to increase logical complexity in inferences with exactly the same content. Since valid deductive inferences depend on logical complexity, differences in neural activity between valid and invalid conditions with the same content could only be attributed to the deductive computational nature of valid inferences. The electroencephalographic (EEG) signals recorded during the experiment are extensively analyzed and compared between both valid deductive and non-valid conditions. Methodologically, the research (i) does not a priori assume the existence of factual differences between logically valid and non-valid deductive inferences, (ii) does not dismiss non-integrable content-independent deductive inferences, and (iii) does not exacerbate the logical complexity of tasks to measure the difference between the neural processing of logical and relational complexity using EEG.

## Material and methods

### Participants

The database was composed by 19 young right-handed subjects (10 males and 9 females), with a mean age of 24.2 years (standard deviation 3.3). One anomalous register was excluded from the final analysis. All participants were recruited during the months of March and May 2019 from the University of León (Spain) and received academic credit for their participation. Participants did not report any significant neurological or psychopathological conditions, or any psychoactive drug intake during EEG recordings. Each participant went through two experimental tasks sequentially. To neutralize eventual learning effects, the invalid condition was first applied to the subjects. First, they performed a LOGICALLY INVALID DEDUCTION paradigm task; afterwards they performed a LOGICALLY VALID DEDUCTION paradigm task. The responding hand for each condition was counterbalanced across subjects. All participants signed an informed consent form before their participation in this study, following the guidelines of the Declaration of Helsinki. The project was approved by the University of León and received the approval of the Ethics Committee (the code of ethics for research is 0-181, dated 11-06-2019). After the pandemic stop, raw data have been analyzed in a trial-by-trial approach at the University of León (Spain).

The experiment was programmed and administered using E-PRIME software. The screen has a sampling rate of 60 Hz, and a resolution of 1024 × 768 pixels. Items were presented against a black background. In both tasks, the index fingers of both hands pressed the keys on a computer keyboard to answer. Participants were sitting 60 cm in front of the screen in a quiet dimly lit environment.

### Experimental design paradigm task

The experiment contrasts two inferential tasks which contain the same stimuli (*i.e.* the same relational variables with the same content and properties), the same premises (relevant cards’ features: figure, color, number and filling) and the conclusion in both tasks is deductive (no new contents beyond the premises are used). The logically valid task applies an explicit rule with logical operators (AND, OR, NOT, IF), while the invalid task is a search task on the contents.

In the invalid task, the subject does not receive any specific deductive rule; instead, they are shown a set of visual stimuli and informed about the cards’ features (figure, color, number, and filling). The instructions for the invalid task were: “If an item follows a rule based on color, figure, number, or filling, press the ‘Ctrl’ key, otherwise press the space-bar”. Thus, the invalid task manages cards’ featured contents without introducing new elements. The invalid task is deductive in the sense that no additional information to the contents given in the premises are used to infer conclusions. Moreover, the invalid task inference is not designed to preserve neither truth nor certainty. Examples of this uncertain or logically invalid deduction are common in the reasoning literature, such as (Feeney, Aidan; [47]).

In the logically valid task, subjects must validly deduce their answer given the definition of what makes up a SET after being shown a trio of cards (*i.e.* an item) with the same contents as in the invalid condition. The logical properties of a SET allow one to determine by logical deduction if any given trio is (or is not) a SET, exclusively applying tools from propositional elementary logic. Any given trio is a SET if all the cards have two or more properties in common. The deductive instruction is: “Press the ‘Ctrl’ key if the presented trio is a SET, otherwise press the space-bar”.

The deduction of the answer (is a SET/is not a SET) is stated without any previous training. It is an integrable inference in all trials, since premises contain all the predicates used in conclusions (see Introduction) and crucially depends on the definition of SET, hence excluding any non-deductive heuristics. It is essential to recognize that the valid task, even if it is simple, has a non-null logical complexity. In summary, the experimental design presents two tasks with the same relational complexity (same viso-semantical content and same relevant categories) but distinct logical complexity (logical operators). Following Friston [60], the content-complextity of a neural task (they call it “cost”) is formalized as mutual information between hidden sates (i.e. perceptual categories) and sensory outcomes (stimuli) under each task condition. A formal proof is not needed to confirm that both conditions in this paradigm have broadly the same informational content. In fact, only in the valid condition there is also certain measurable logical complexity given by the propositional operators defining SET. The experiment tries to demonstrate or refute neuroelectrical EEG measurable differences between logically valid inferences based on logical operators and not logically valid inferences based on visosemantical content.

### Stimuli

The items in the study were trios of cards from the game SET (Set Enterprise, 2019). Each card has a variation of the following four features: figure (diamond, ovoid, or squiggle), color (green, red, or black), cardinality (1 or 2), filling (filled or empty). None of the participants was familiar with the game or its rules. The experiment included the same 200 randomly ordered trials in both conditions. Presenting the same stimuli ensures that the relational complexity of both conditions is exactly the same; that is, they include the same set of cards and are described with the same nomenclature and lexical card descriptions. On the other hand, instructions for the valid condition (SET definition and logical constants) ensure that the valid task has a measurable logical complexity.

**Fig. 1:**
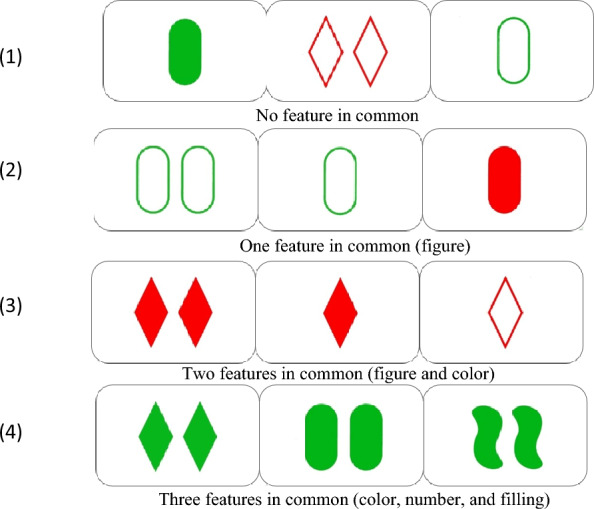
Example of items of SET in both conditions: valid and invalid deductions.

Any given trio of cards either does or does not share the same relevant features: figure, color, number, and filling. Figure 1 displays examples of trios which do not share any features (case 1), share one feature (case 2), share two features (case 3), or share three features (case 4).

Both valid and invalid tasks are ecological and user-friendly since the paradigm is inspired and presented as a game. The design does not allow the researchers to describe the precise inference pattern followed by any subject in any trial. For cases (3) and (4) (see Fig. 1), positive propositional inferences are enough, particularly connectives and the Modus Ponens rule (deduce B from {A, if A then B}). Cases (1) and (2) can be negatively treated with connectives and the Modus Tollens rule (deduce not A from {not B, if A then B}). The point of the experiment is not to follow the neural processing of any specific pattern, but to study any deductively valid inference.

### Time chart

**Fig. 2:**
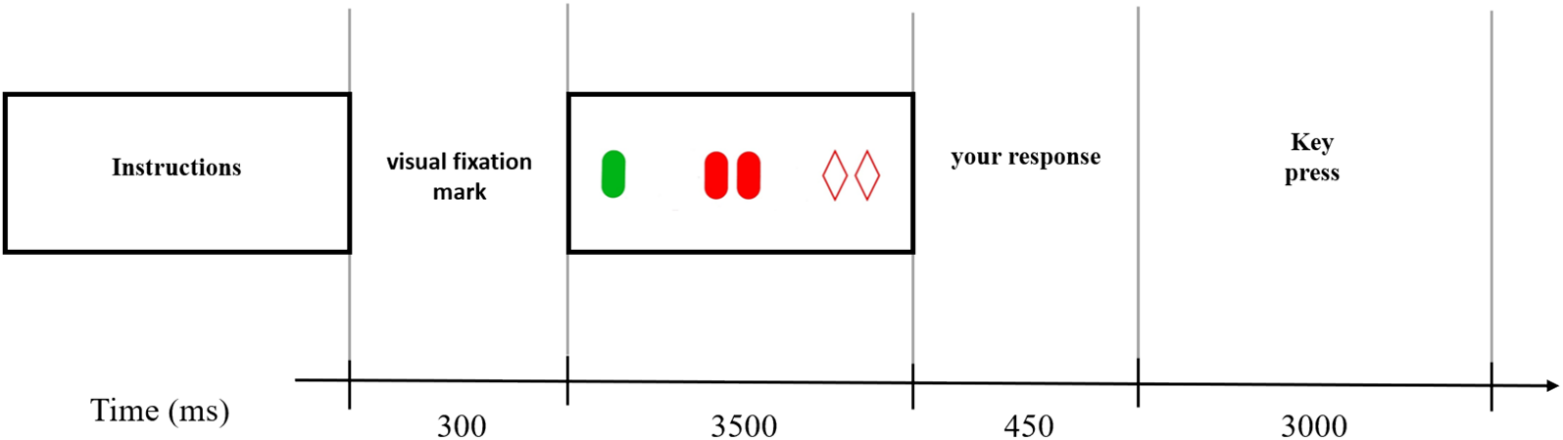
Timeline of stimuli in milliseconds for a generic trial

The beginning of the trial was signaled by a cross ( +) presented in the center of the screen for 300 ms, which was then followed by the appearance of the items on the screen for 3500 ms. Then, the items disappeared, and the central dot reappeared for 450 ms. Participants were asked to respond quickly (within 3000 ms). The time chart is presented in Fig. 2.

### Recording and preprocessing of EEG signals

The EEG was recorded with a 64-channel amplifier (Neuronic System, Cuba) and specific acquisition software (Neuronic EEG/Edition EEG Software) with a sampling rate of 200 Hz. Reference electrodes were placed on the earlobes. In addition, electrooculography (EOG) signals were acquired using three pairs of sensors in order to acquire the horizontal and vertical movement of the eyes. Electrode impedance was kept below 5 kΩ. Extracephalic channels were removed for the subsequent analyses thus keeping 58 EEG channels according to 10–10 system:: FP1, FP2, F3, F4, C3, C4, P3, P4, O1, O2, F7, F8, T3, T4, T5, T6, FZ, CZ, PZ, F1, F2, P1, P2, AF3, AF4, P5, P6, FC5, FC6, C5, C6, TP7, TP8, PO7, PO8, FPZ, FCZ, CPZ, POZ, OZ, PO3, PO4, CP1, CP2, CP3, CP4, C1, C2, F5, F6, FC3, FC4, FC1, FC2, CP5, CP6, TP9, and TP10. Furthermore, EEG channels were grouped in 13 regions of interest (ROIs) according to Table 1.

**Table 1:** Correspondence between ROIs and EEG channels

Channels	ROI
FP1, FP2, AF3, AF4, FPz	Prefrontal
F4, F8, FC6, F6, FC4	Right frontal
F3, F7, FC3, F5, FC5	Left frontal
Fz, F1, F2, FCz, FC1, FC2	Medial frontal
T4, T6, TP8, TP10	Right temporal
T3, T5, TP7, TP9	Left temporal
C4, C6, CP4, CP6	Right central
C3, C5, CP3, CP5	Left central
Cz, CPz, CP1, CP2, C1, C2	Medial central
P4, P6, PO8, PO4	Right parietal
P3, P5, PO7, PO3	Left parietal
Pz, P1, P2, POz	Medial parietal
O1, O2, Oz	Occipital

The preprocessing consisted on 4 steps: (i) application of bandpass (1–70 Hz) and notch (49.8–50.2 Hz) Finite Impulse Response (FIR) filters with a Hamming window to limit noise bandwidth and to remove powerline noise, respectively; (ii) artifact rejection by means of independent component analysis (with special care to remove eye-derived artifacts, see Additional file 1: Figures S1 and S2); (iii) selection of 1.5-s *useful* trials; and (iv) thresholding to remove noisy trials [61]. The *useful* trial selection consisted of localizing a stimulus followed by a correct response and another stimulus, thus discarding stimulus with more (or less) than one response. The trial length was 1.5 s comprising two intervals: 0.5 s before the stimulus, acting as baseline, and 1 s after the stimulus. Furthermore, the trials whose response time (*i.e.* time elapsed between the stimulus and the response) was less than 1 s were discarded to minimize the influence of the motor responses in the event-related potentials (ERPs).

### Evoked potentials: synchronized averaging of trials

Firstly, the evoked potentials were analyzed to study the electrophysiological response of both experimental deductive conditions (valid and invalid). For this task, all the trials of each condition were averaged to study P3, N4, and P6 components, as they are related with deductive processing (premise integration in P300, semantic analysis in N400 and second processing in P600). Furthermore, the topographic distribution of the potentials was also analyzed.

### Time–frequency analyses: trial-by-trial approach

The properties of EEG signals are not stationary but they vary over time [62]. Thus, methods as Fourier transform that require stationarity should not be used to analyze the time-varying properties of ERPs. In line with that, continuous wavelet transform (CWT) provides a framework to study the dynamical properties of the frequency content of the ERP signals. A wavelet is a function with zero-mean with a localization in time and frequency [63]. We have chosen as mother wavelet the complex Morlet, as it has been proven to provide a good fit with biological data [61]. To carry out the analysis, the complex Morlet is dilated and translated to generate a wavelet family that is able to capture the fast time-varying properties of the signal with a high frequency resolution [64]. The dilation factor sweeps from 1 to 70 Hz with equally spaced intervals of 0.5 Hz [61]. Wavelet analysis offers a solution to analyze signals with a high temporal resolution while keeping the frequential resolution also high [64]. It is possible because of its variable time–frequency resolution, with shorter time windows used for higher frequencies and longer windows used for lower frequencies. Deeper insights about wavelets can be found in [64].

From the wavelet decomposition obtained after applying the previous analysis, the wavelet scalogram was computed for each ERP trial. Then, wavelet coefficients were squared and normalized by the baseline, thus converting them into normalized wavelet power coefficients. This normalization is employed to unveil event-related dynamics that otherwise may remain concealed [65]. They summarize the power distribution of the wavelet in the time–frequency plane. This time–frequency representation of the energy of each trial can be used to identify the spectral content associated to specific frequency ranges. In this study, we have considered the conventional EEG frequency bands: delta (*δ*, 1–4 Hz), theta (*θ*, 4–8 Hz), alpha (*α*, 8–13 Hz), beta-1 (*β*_1_, 13–19 Hz), beta-2 (*β*_2_, 19–30 Hz), and gamma (*γ*, 30–70 Hz).

### Statistical analysis

Data distributions were tested in an exploratory analysis to assess normality and homoscedasticity. The first one was tested using Lilliefors test, while the latter with Bartlett test. Response time distributions, peak amplitude distributions, ERPs and wavelets did not meet the normality and homoscedasticity hypotheses; therefore, Wilcoxon signed rank tests were used to assess differences between valid and invalid conditions. Furthermore, to assess the correlation between the time courses of the evoked potentials, Spearman tests were employed, as this method can detect both linear and nonlinear correlations.

### Data availability

Scripts to calculate wavelets including raw and preprocessed data are available in: Víctor Rodríguez, Francisco Salto, Carmen Requena (2022), “Invalid and Valid Deductive Processes”, Mendeley Data, V2, https://doi.org/10.17632/w95n6rc6fs.2

Victor Rodriguez. (2022). < i > Invalid and Valid Deductive Processes < /i > [Data set]. Mendeley. https://doi.org/10.17632/GM49MM6WHW.1

Victor Rodriguez. (2022). < i > Invalid and Valid Deductive Processes < /i > [Data set]. Mendeley. https://doi.org/10.17632/W95N6RC6FS.2

## Results

### Behavioral data

Response time (RT) was obtained for each subject and condition within a temporal window of 3 s. Statistically significant higher RTs were observed for the valid deductive condition than for the invalid condition (*p*-value < 0.0001, Wilcoxon test; see Table 2).

**Table 2:** Descriptive data associated with response time for each condition: invalid deductive and valid deductive

	Valid condition	Invalid condition
Mean (ms.)	2890.35	1977.84
Median (ms.)	2660.00	1705.00
Standard deviation (ms.)	1368.59	917.89

In the logically valid condition, the subjects answered correctly 96.14% of the trials. The incorrect responses were not considered in the analysis. In the invalid condition, there were no evaluable correct or incorrect answers.

### Event-related potential data

**Fig. 3:**
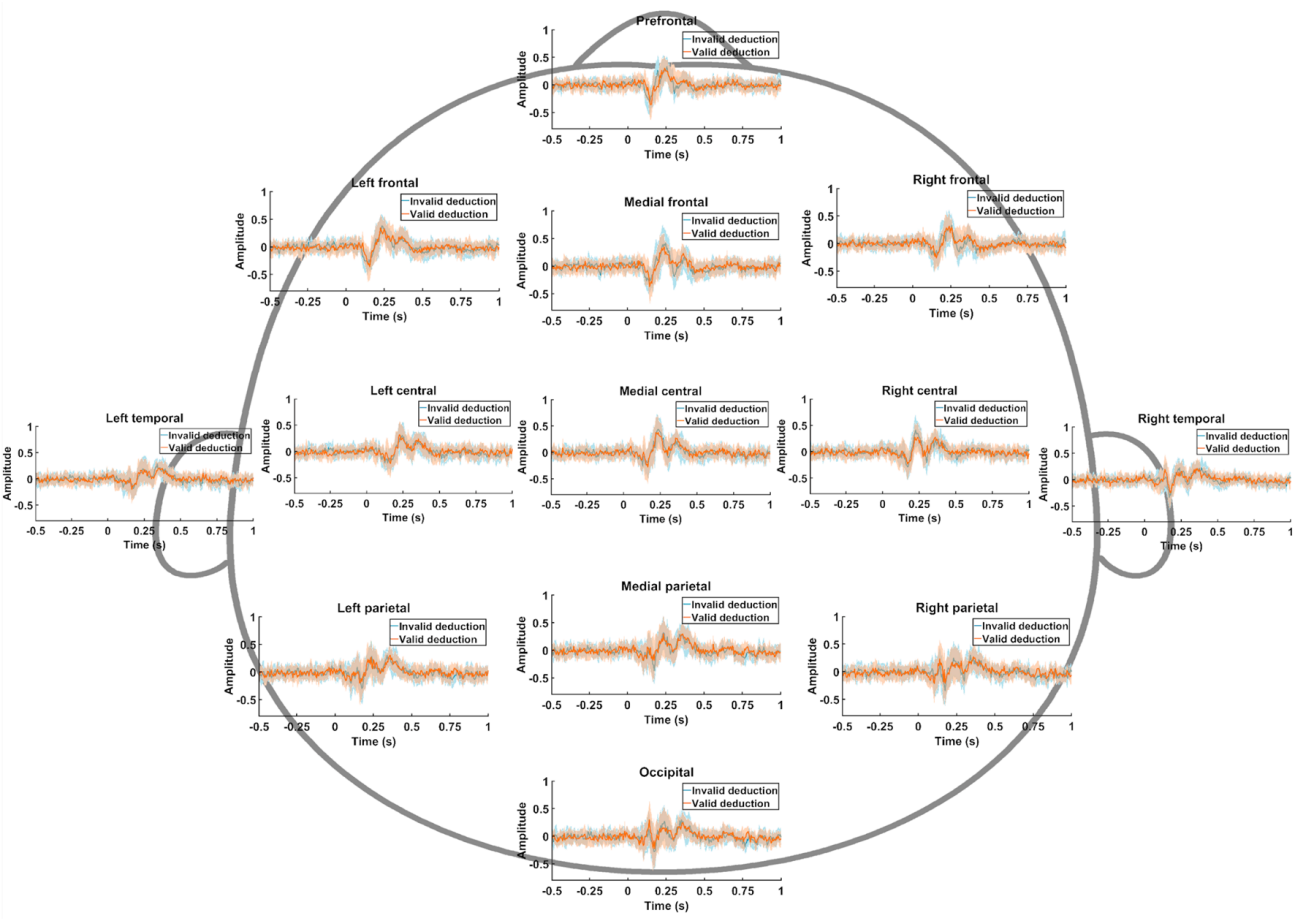
Averaged time courses of the ERPs for all the ROIs and conditions

Figure 3 shows the averaged time courses for all the ROIs and conditions. For each ROI, the potentials associated with valid deductive and invalid inferences are depicted simultaneously to ease the comparisons between them. Remarkably, the temporal evolution of both invalid and valid deductions is very similar. Their correlations were tested, showing a mean correlation value of 0.30 (*p* < 0.05, Spearman correlation test). In the time courses of the evoked potentials, four (positive or negative) peaks are identified likely corresponding to N250, P300, N400 and P600 potentials for both valid and invalid conditions. Since N250 is an attentional component identical in both conditions, it has not been included in the analysis. At some points, a deviance could be appreciated between invalid and valid deductive processes (*e.g.,* right frontal ROI around 300 ms). Nonetheless, no statistically significant differences were found when evaluating both peak and mean amplitude in the potentials P300 (220–250 ms), N400 (300–350 ms), and P600 (500–550 ms).

**Fig. 4:**
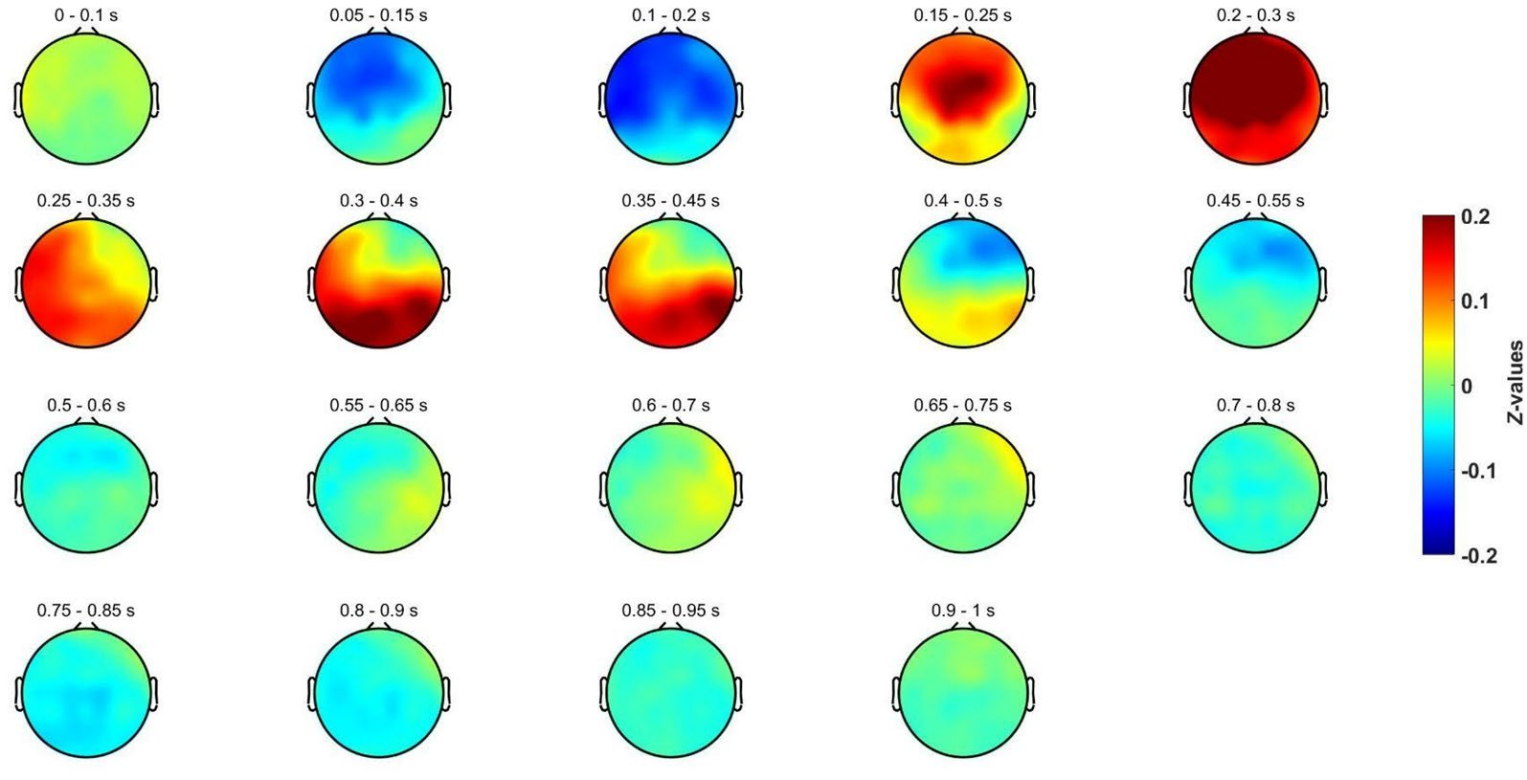
Spatial distribution for the baseline-corrected evoked potentials for the invalid deductive condition using a sliding window of 0.1 s

**Fig. 5:**
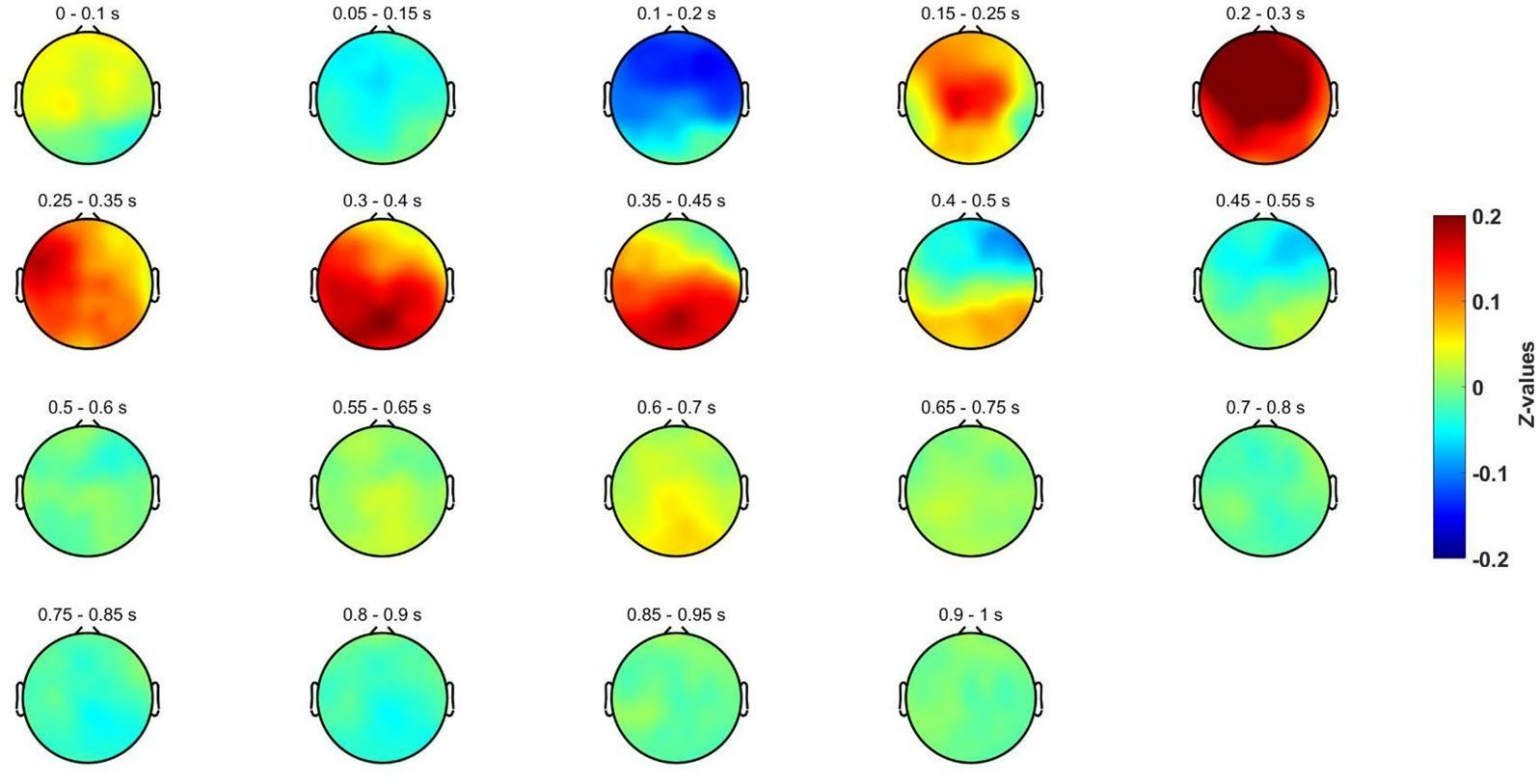
Spatial distribution for the baseline-corrected evoked potentials for the valid deductive condition using a sliding window of 0.1 s

**Fig. 6:**
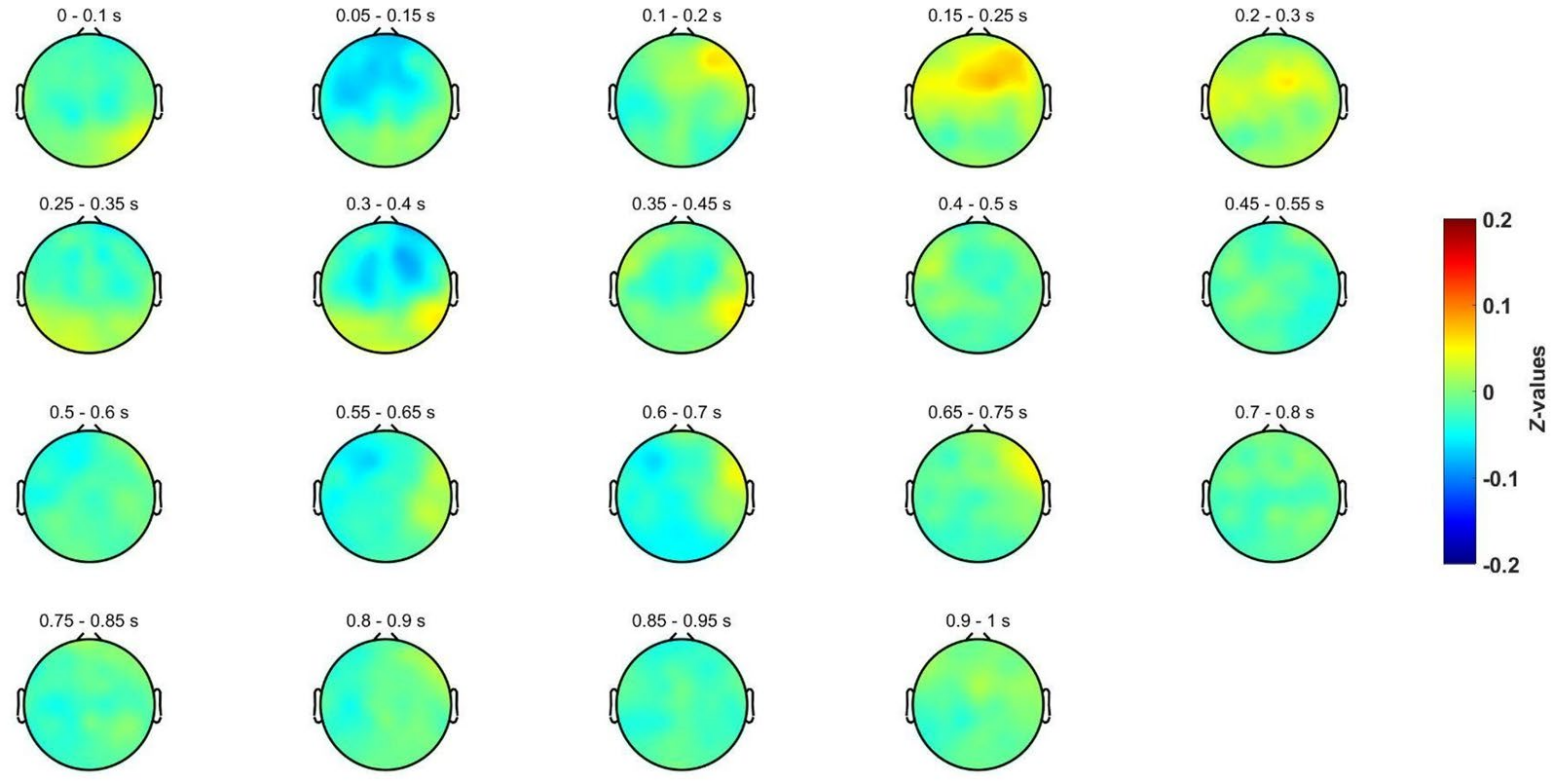
Spatial distribution for the baseline-corrected evoked potentials for the difference between invalid and valid deductive conditions using a sliding window of 0.1 s

Other perspective of the time courses of the evoked potentials is provided in Fig. 4, and Fig. 5. In them, it could also be appreciated the evoked potentials (N250, P300, N400 and P600) that occur in a similar way for both conditions. Nonetheless, in some ROIs and time intervals, a deviant behavior was found around 400 and 600 ms. Here, it is also relevant Fig. 6, which depicts the difference between both invalid and valid deduction in each time interval. Observing these differences yielded us to conduct more detailed analyses in order to isolate the brain patterns that provoke them.

### Time–frequency analysis

In Additional file 1: Figure S3 it is depicted the time–frequency activation for the different conditions (logically invalid and logically valid). It is shown that the activation process associated to the experiment mainly occurs in slow bands (delta and theta) from 0.25 s on. No observable differences can be appreciated between the time–frequency activations between invalid and valid conditions.

**Fig. 7:**
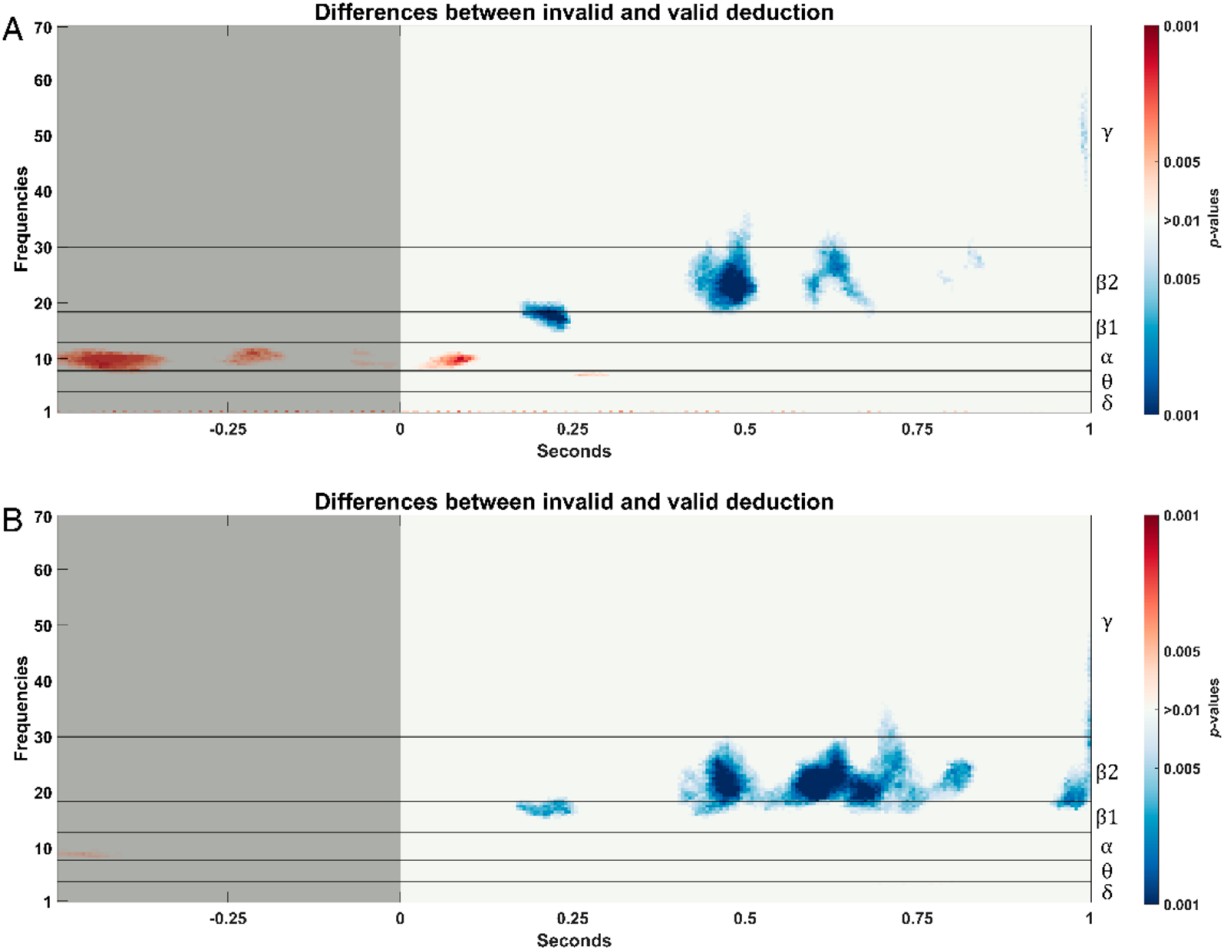
Statistically significant differences (p-values < 0.01, Wilcoxon signed rank test) found between logically invalid and logically valid conditions for: **A** left temporal; **B** left central. Baseline period has been shaded. In left axis, the conventional EEG frequency

To evaluate whether is it possible to find differentiating patterns between conditions (logically invalid and logically valid), we repeated the time–frequency analysis focusing on the ROIs involved in deduction processes. We calculated the wavelet activation for each subject, ROI, and condition. Afterwards, to look for differentiating patterns, we performed an exploratory statistical analysis by comparing them for each time–frequency bin. The results of this analysis are depicted in Fig. 7. As no statistical correction was applied due to the high number of time–frequency bins, these results can be considered exploratory. Nonetheless, it could be appreciated that some statistically significant differences arose in left central and left temporal regions around 400 and 600 ms.

**Fig. 8:**
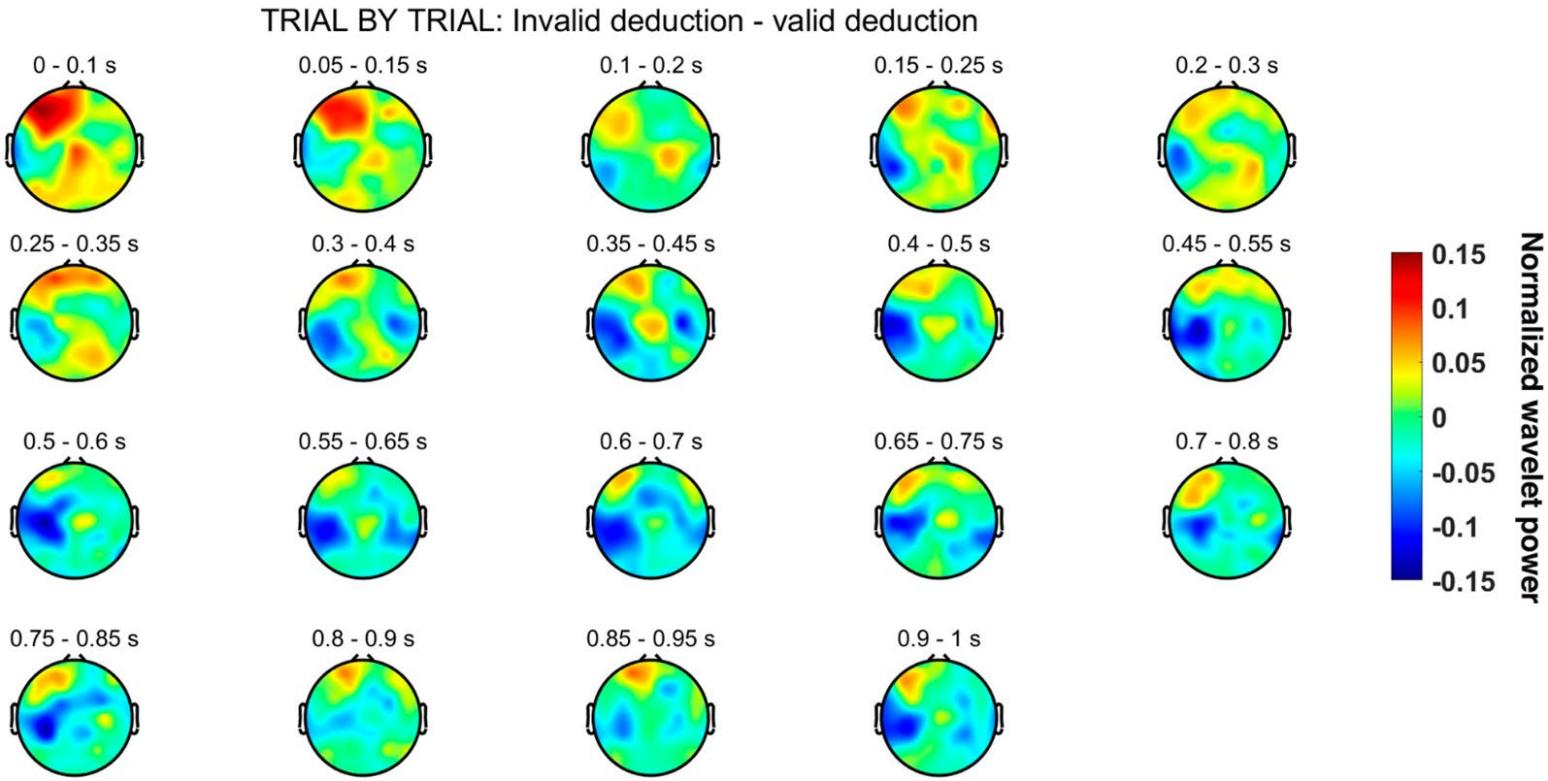
Spatial distribution of the difference of the wavelet activation in beta-2 between invalid and valid conditions using a sliding window of 0.1 s

Finally, the topographical distribution of the patterns that shows a deviance between both conditions was assessed. In Fig. 8, it is depicted the difference between logically invalid and valid conditions for different time windows. Of note, these patterns correspond with the trial-by-trial time–frequency analysis in beta-2, where these differentiating patters can be appreciated. It can be observed that this deviant behavior mainly occurs around the left temporal region between 400 and 600 ms.

## Discussion

In this study, we introduce a new paradigm that compares the neural activity of logically valid versus invalid deductive inferences with precisely the same content (same premises with the same relational variables) and different logical complexity. The evoked response of logically valid and invalid conditions follows a similar neural pattern and latencies. Nonetheless, a fine-grain trial-by-trial analysis shows significant differences in the time–frequency activation of beta-2 band in left central and left temporal areas after 400 ms (*i.e.*, after premise integration phase [15, 24, 26, 27] and after 600 ms (*i.e.*, reprocessing phase [16, 66]). Logically valid and invalid conditions present congruent deflations at N250 (perceptual task), P300 (premise integration), and N400 (semantical content). However, the logically valid deductive condition presents specific electrical features: (i) increased beta-2 band activation at initial (400 ms.) and late (600 ms.) phases in central and left temporal areas, and (ii) slower reaction times (61.54%). These findings suggest that semantical content mostly but not completely determines all the inferential brain electrical activity, since processing of logical complexity in the valid condition involves subtle but measurable temporal and time–frequency differences.

(i) Same evoked potentials and same topography: Logically Valid and Invalid deductive inferences with the same content evoke the same response pattern

Evoked potentials in both conditions include the same positive deflection at 250 ms (perceptive recognition of stimuli) [67], as well as the same P300 and N400 potentials (see Figs. 3, 4). P300 has been extensively associated with premise integration both by the neuroelectric literature [28–31] and by magnetic resonance research [20]. Its presence is explained by the fact that both conditions are equally integrable (they share all relational variables). The congruence in the potentials evoked by valid and invalid inferences (Figs. 3, 4) can be explained because both tasks have the same content, namely the same relational variables from the same SET cards with the same relevant properties in both conditions. In this regard, it is content and not logical form that determines the evoked patterns produced by the neural processing of these inferences, both logically valid and invalid. This fact is particularly relevant for the understanding of deductive reasoning and coincides with other non-formalistic results [2, 47] in which semantical content (and not logical form) determines not only invalid but also valid inferences [33, 68, 69].

Moreover, the initial topographical analysis does not show noticeable significant differences between logically valid and invalid inferences (See Figs. 4, 5 and 6). These results do not support with EEG data any specific core region of deductive processing. In particular, left frontal areas [33] and cingulo-opercular regions [19], typically associated with semantic processing, are similar in both conditions, which is reasonable as they depend on the same visual content also in both conditions. In other neurophysiological studies on inferential tasks, the cortical topography did not show relevant changes either, as it is the case in [70], where reasoning and attention are equivalent.

(ii) Forward Inference Methodology: Sensitivity and validity of the paradigm

Forward methodologies for the neural study of inference have been explicitly defined by Heit [71] to associate cognitive inferential processes with neural events. Any hypothesis stating that the same cognitive process underlies logically valid and invalid inferences is refuted by neural differences in (regions)x(potentials)x(frequencies) between the two conditions. In this paper, we infer that the same cognitive processing is not supported because of slight differences found in beta-2 in two specific ROIs.

Statistically significant differences (*p*-values < 0.01) were found in left temporal and left central regions for beta-2 oscillations. By forward inference [71], the valid condition corresponds to a distinct frequential pattern suggesting a distinct cognitive process. The specific temporal location of this beta-2 activity verifies the adequacy and sensibility of the experimental design, since only a strong coherence among time-dependent ERP trials explains the specific temporal location of beta-2 band activity [72].

Logically valid inferences are homogeneous in their electrical features in cases (1), (2) just as in cases (3), (4). Even if reasoners in the logically valid condition are free to choose the order of premises and logical operators, the neuroelectrical responses are homogeneously late, slow-responsive, time-extended, and differentially accompanied by beta-2 oscillations.

(iii) Beta-2 oscillations in logically valid inferences

The beta-2 activation occurs consistently in both left areas (see Figs. 7, 8) where it appears in two distinctive time intervals: (i) around 400 ms, and (ii) around 600 ms. Moreover, the differences in beta-2 oscillations are exclusively found in these time intervals and these two left brain areas. The early literature [73] already associated beta band with logical tasks, and ulterior research has verified its presence in top-down cognitive control [74], cognitive load [75], grammar [76], and false reasoning [77]. Even if a comprehensive theoretical explanation of the role of beta-2 in deductive reasoning is still lacking [78, 79], neurocomputational research has shown its role in the maintenance of repeatable or abstract properties in the same information (Bernhard [80, 81]. Limanowski and Friston found sensory/cognitive differential interaction effects in beta also related to rule-following instructions [82] and recent MEG [83] and EEG (Álvarez-Merino, 2019) research associate beta-2 band activity with logical reasoning in the early premise integration phase (300–350 ms) and again later in the re-processing phase (600–650 ms). Two salient features in the temporal and spatial distribution of beta waves are the following:

- The first beta-2 differences are temporally located just after the premise integration phase (see Figs. 7, 8). Since the content of premises in both conditions is exactly the same and, moreover, it is integrable (contents are shared by premises and conclusions), the differences in beta-2 oscillations must be linked not with content but with logical complexity, that it is only explicitly present in the valid condition. Beta oscillations accompanying positive (negation free) logical operators (conjunction, disjunction, material conditional) have been reported in the literature in semantical [84] and visuo-semantical contexts (Álvarez-Merino, 2019). Two other experiments with the same SET paradigm applied in MEG studies on amplitude [83] and connectivity (to appear) found similar beta-2 activations at the early stages of the deductive inferential process.
- The second time region of differences in beta-2 (see Figs. 7, 8) is a late oscillation accompanying the “second processing” typically present in inferences with propositional operators [16]. As for the previous differences, they are probably provoked by the different logical complexity between conditions. The time extended duration (400-650 ms) of the differences in beta-2 activity between the two conditions, as well as its late beginning, is not to be attributed to the memory charge or the relational complexity of the task (as in [75], since they are the same in both conditions. It is plausible to interpret beta-2 activity in the second processing not only as control and monitorization [74, 78], but as the result of a specific logically valid computation. Significantly, the role of cognitive beta band oscillations has been verified in rule selection [84] and both beta-2 activation are consistent with rule-determined computations in the valid condition.
- Spatial distribution of beta-2 oscillations in logically valid inferences.

The spatial limitations of EEG measures notwithstanding, there are important facts about the spatial distribution of beta-2 in the left central and left temporal regions since its presence throws some light on the cortical relationships between language and valid deductive inference. Visual inferences in both experimental conditions are processed fundamentally in the left hemisphere, as some other cerebral studies on deduction have shown [24, 33, 83]. In this study, there is no electrical evidence of deductive core areas in the valid condition, but beta-2 oscillations coincide with opercular and triangular activity described by deductive meta-studies [19]. Significantly, left frontal and parietal regions do not show statistically significant differences between the two conditions (See Additional file 1: Figure S4). Previous studies have associated increased beta-2 activity and connectivity with logically valid inferences [83] also in opercular and triangular areas. There are no differences in theta band activity between conditions, suggesting their similar visuo-semantical processing. These results confirm the multiple functionalities of opercular circuits in linguistic and visual inferences [33], as well as the involvement of the same opercular areas in linguistic and deductive tasks, but with specific beta-2 activation in deductive valid inferences. Since medial theta band activity has been associated with semantical content (Duprez, Gulbinaite, & Cohen, 2020) the absence of differences in theta band suggests that the presence of beta-2 is linked not with semantical content (in this case visuo-semantical) as in [54] and [85], but with logical operators.

(v) Recursive processing hypothesis

The fact that reaction time in the logically valid condition is 61.54% slower, is not to be explained by general control processes as they also apply to the invalid condition that has the same content. The literature on the neural correlates of propositional reasoning has identified and studied this kind of late re-processing in linguistic and visual formats [16]. interpret late re-processing in propositional logically valid inference by means of mental model semantics. [52, 53], interpret similar late propositional re-processing not by semantical processing, but by logical complexity. The electrophysiological results in this paper don’t settle the question but confirm a similar spatial distribution of cortical deductive activity in visuo-semantic and logical processing and a slight frequential divergence between semantic and logical processing [20, 33]. Logically valid inferences are (i) homogeneously late (see Fig. 7), (ii) time-extended (see Fig. 8), (iii) slow-reactive (see Table 2), and (iv) with noticeable beta-2 presence in left central and left temporal regions (see Fig. 7). Is there a common explanation to these apparently opposing features?

A plausible answer is the recursive nature of valid deductions as cortical events. The literature has associated automatic rule-following processes with exactly the kind of beta-band hypoactivity we find in valid deductions [74, 86]. In the case of logically valid proofs of the elementary kind involved in SET inferences, if automaticity is understood as the ability to perform a routinized task with minimal effort [87], logically valid deductions manifest themselves as slow automatable processes. In fact, logically valid deductions, as abstract objects, are demonstrably recursive or computable [6, 88], and as slow, hypoactive and homogeneous cortical events, they may be understood as explicitly routinized automatable neural processes. Thus, the remarkable delay in the valid deductive processing can be explained by the recursive nature of valid inferences, which are in fact, unlike invalid inferences with the same content, deductive proofs of SET properties. Notice that recursive computational procedures are faster and more efficient than simple iterations [89], but they are still slow compositional processes based on strict routines and they exclude abbreviated procedures or heuristic jumps. Consider for example Modus Ponens deductions (deduce B from {A, If A then B}) in visual [90] and motor [91] contexts. In SET, recursive computations correspond to step-by-step procedures or proofs that are consistent with retarded and automatable processes. The paradigm does not allow us to determine which specific sequence is followed in the valid condition, but we know that *some* kind of valid ordered sequence of SET rules is successfully followed. Other EEG studies are consistent with understanding valid logical deductions as slow automatisms, given their higher mean duration [92].

## Conclusion

This study presents some limitations regarding its sample size, the exploratory nature of the statistical analysis and the spatial resolution of the EEG techniques employed. Future research with this paradigm will introduce an additional perceptual baseline to control eventual instruction effects. However, the new experimental paradigm proposed in this study is effective and capable of identifying differential neural patterns between logically valid and invalid reasoning without following any specific logical operator. Results in potentials, latencies, amplitudes, and topologies are consistent and point to the absence of any specific EEG discriminable cerebral locus nor potential for logically valid deductions. Content and not logical form determines most of the electrical features of deductive reasoning irrespective of its normative status (logically valid or invalid). However, there are in fact specific cortical features of logically valid inferences in terms of temporal delay and beta-2 late processing after 400 ms and after 600 ms, specifically in the valid condition and left temporal and central areas. These frequential differences are sufficient to consider logically valid deductions as distinct neural processes. It is hypothesized that the recursive nature of logically valid deductions explains the electrical relevance of logical validity. The evidence offered shows that certain neuroelectrical conditions are sufficient for logically valid deductions, but it does not show that they are necessary.

## Supplementary Information

**Additional file 1**: **Figure S1**. Correlation values of valid deductive signals beforeand afterthe application of ICA. The first row corresponds with the horizontal EOG channel, the second one with the right vertical EOG channel, and the third row with the left vertical EOG channel. **Figure S2**. Correlation values of invalid deductive signals beforeand afterthe application of ICA. The first row corresponds with the horizontal EOG channel, the second one with the right vertical EOG channel, and the third row with the left vertical EOG channel. **Figure S3**. Grand- average of the time-frequency representations of invalid deductive and valid deductive. The representations have been normalized by the baseline meanin each time point. Baseline has been shaded. In right axis, the conventional EEG frequency bands have been delimited and tagged. **Figure S4.** Statistically significant differencesfound between invalid and valid conditions for: A): left frontal; B): left parietal. Baseline period has been shaded. In left axis, the conventional EEG frequency bands have been delimited and tagged. Red points indicate statistically significant differences for which invalid deductive has more power, while blue differences represent time-frequency bins in which valid deductive process obtained higher power than invalid one.

## Data Availability

*Víctor Rodríguez, Francisco Salto, Carmen Requena (2022), “Invalid and Valid Deductive Processes”, Mendeley Data, V2, *https://doi.org/10.17632/w95n6rc6fs.2* Victor Rodriguez. (2022).* < *i* > *Invalid and Valid Deductive Processes* < */i* > *[Data set]. Mendeley. *https://doi.org/10.17632/GM49MM6WHW.1* Victor Rodriguez. (2022).* < *i* > *Invalid and Valid Deductive Processes* < */i* > *[Data set]. Mendeley. *https://doi.org/10.17632/W95N6RC6FS.2.
