# Supplementary material for: Electrical analysis of logical complexity: an exploratory eeg study of logically valid/invalid deducive inference
Source: Brain Inform. 2023 Jun 7;10(1):13. doi: 10.1186/s40708-023-00194-8 (PMC10247637; doi:10.1186/s40708-023-00194-8)
Supplement: Supplementary file 1 — Additional file 1: Figure S1. Correlation values of valid deductive signals beforeand afterthe application of ICA. The first row corresponds with the horizontal EOG channel, the second one with the right vertical EOG channel, and the third row with the left vertical EOG channel. Figure S2. Correlation values of invalid deductive signals beforeand afterthe application of ICA. The first row corresponds with the horizontal EOG channel, the second one with the right vertical EOG channel, and the third row with the left vertical EOG channel. Figure S3. Grand- average of the time-frequency representations of invalid deductive and valid deductive. The representations have been normalized by the baseline meanin each time point. Baseline has been shaded. In right axis, the conventional EEG frequency bands have been delimited and tagged. Figure S4. Statistically significant differencesfound between invalid and valid conditions for: A): left frontal; B): left parietal. Baseline period has been shaded. In left axis, the conventional EEG frequency bands have been delimited and tagged. Red points indicate statistically significant differences for which invalid deductive has more power, while blue differences represent time-frequency bins in which valid deductive process obtained higher power than invalid one. [file 40708_2023_194_MOESM1_ESM.docx]

Additional file

To assess the ability of our preprocessing to remove eye-related artifacts, the correlation of the channel data with the electrooculographic (EOG) channels was assessed before and after the artifact rejection carried out by means of independent component analysis (ICA). In the figures below it is depicted the correlation of the EEG channels with the different EOG channels, before (left) and after (right) the application of ICA. It can be observed that the correlation of the EEG data with the EOG channels dramatically decrease after the ICA component rejection. Thus, it can be concluded that the influence of the eye artifacts in the preprocessed signals can be neglected.


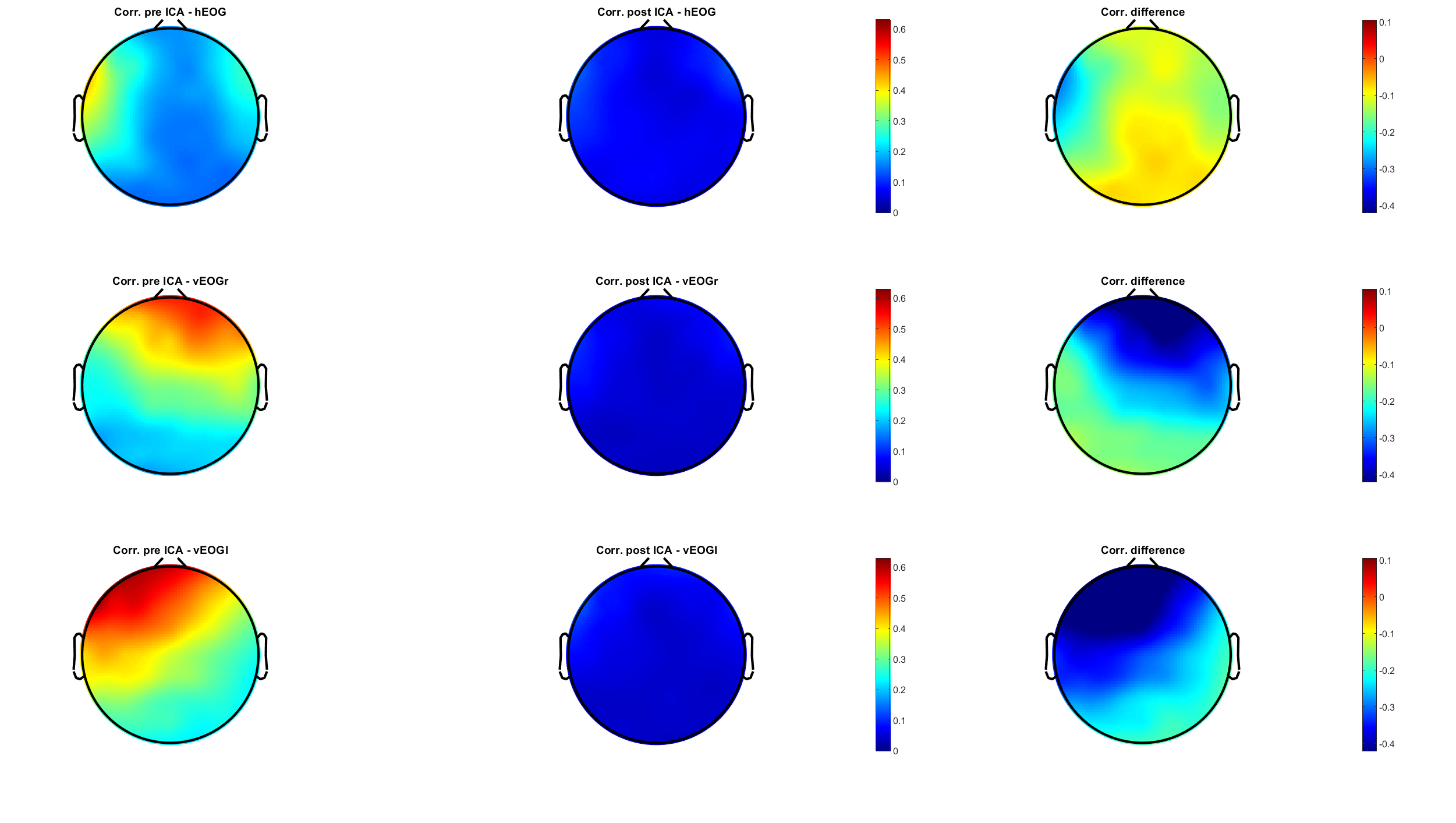


**Figure S1**. Correlation values of valid deductive signals before (left) and after (right) the application of ICA. The first row corresponds with the horizontal EOG channel, the second one with the right vertical EOG channel, and the third row with the left vertical EOG channel.


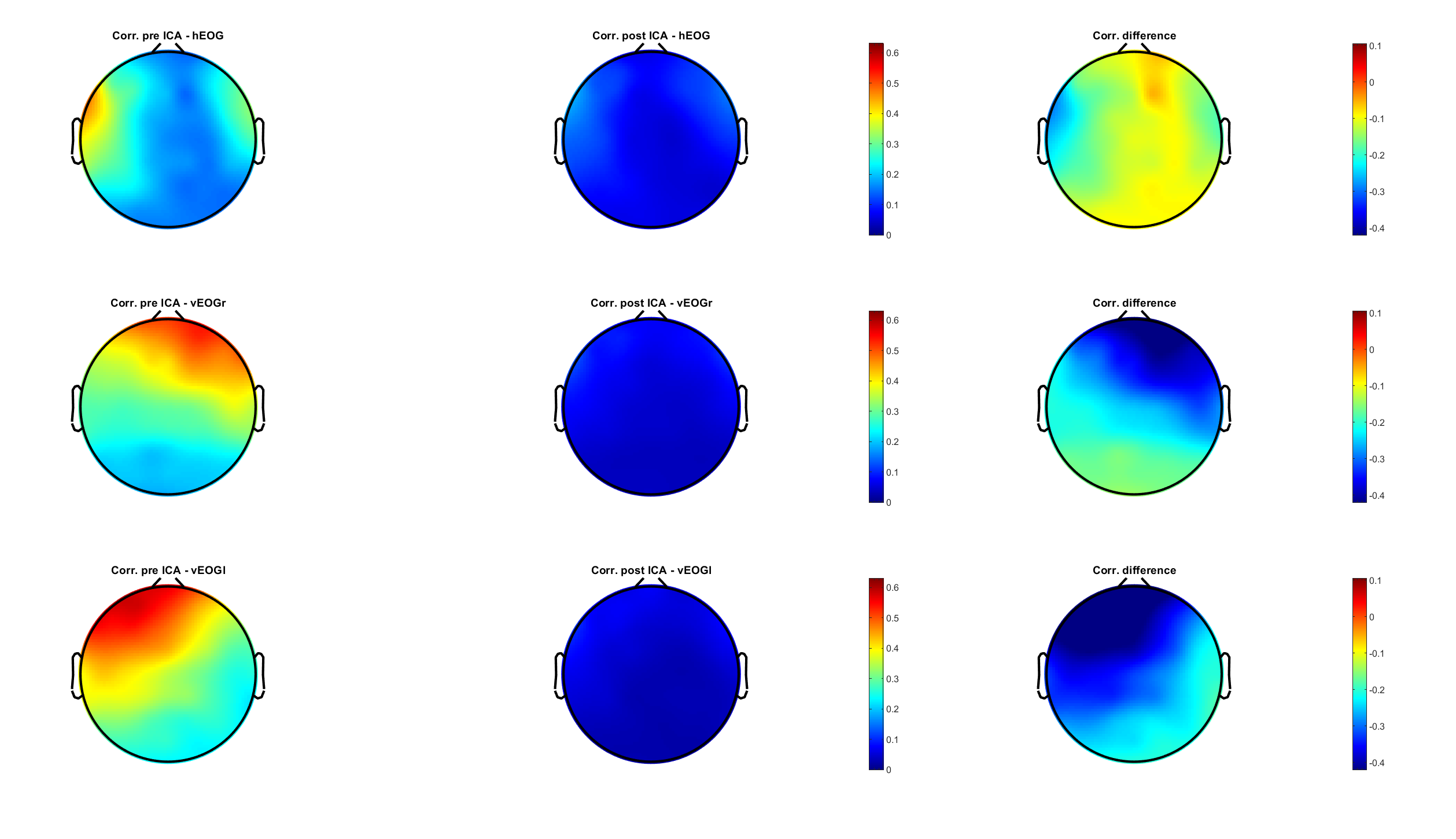


**Figure S2**. Correlation values of invalid deductive signals before (left) and after (right) the application of ICA. The first row corresponds with the horizontal EOG channel, the second one with the right vertical EOG channel, and the third row with the left vertical EOG channel.


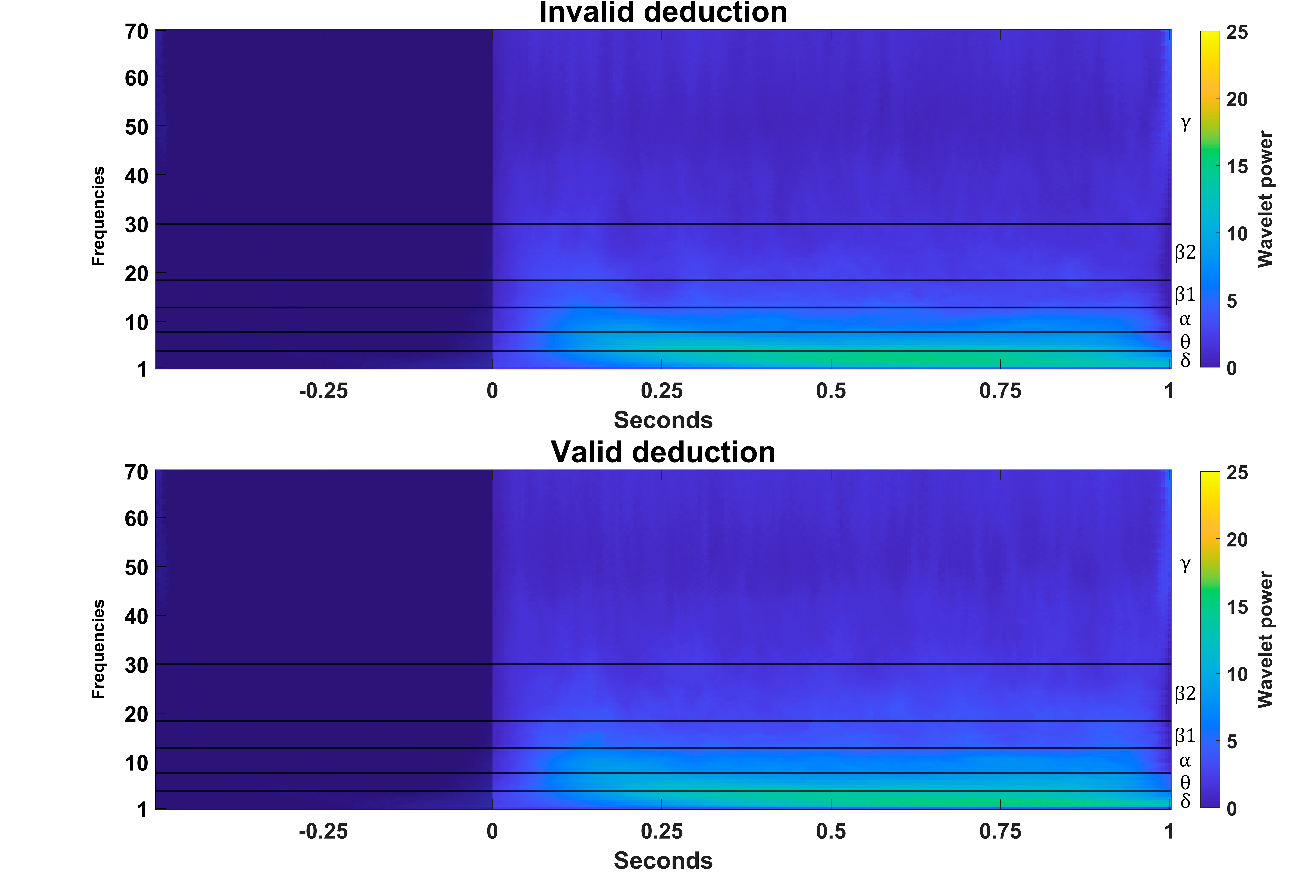
**Figure S3**. Grand- average of the time-frequency representations of invalid deductive and valid deductive. The representations have been normalized by the baseline mean (-500 - 0ms) in each time point. Baseline has been shaded. In right axis, the conventional EEG frequency bands have been delimited and tagged.


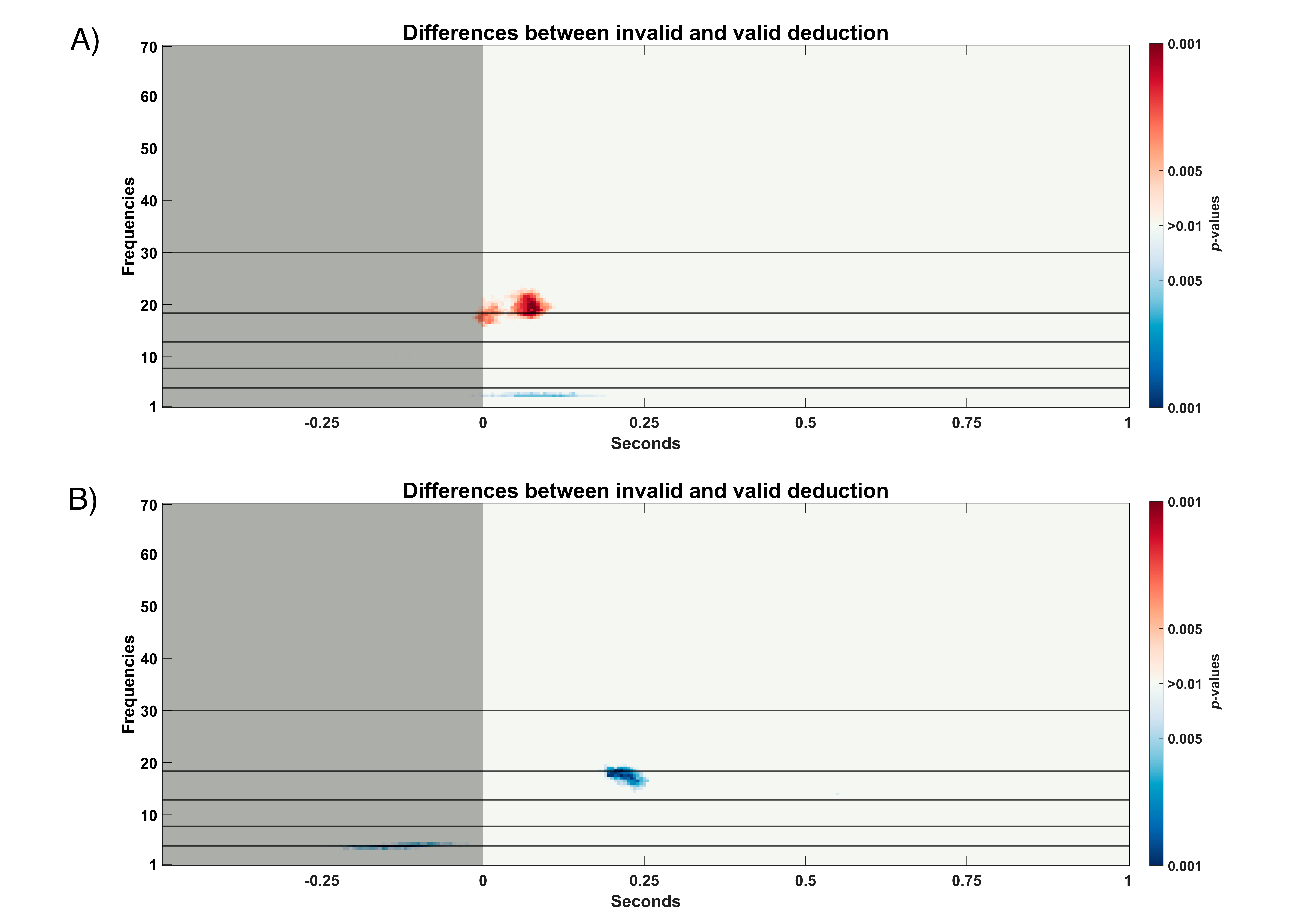


***Figure S4.*** *Statistically significant differences (p-values < 0.01, Wilcoxon signed rank test) found between invalid and valid conditions for: A): left frontal; B): left parietal. Baseline period has been shaded. In left axis, the conventional EEG frequency bands have been delimited and tagged. Red points indicate statistically significant differences for which invalid deductive has more power, while blue differences represent time-frequency bins in which valid deductive process obtained higher power than invalid one.*
